# Supplementary material for: Prevalence of diarrheagenic Escherichia coli and impact on child health in Cap-Haitien, Haiti
Source: PLOS Glob Public Health. 2023 May 5;3(5):e0001863. doi: 10.1371/journal.pgph.0001863 (PMC10162540; doi:10.1371/journal.pgph.0001863)
Supplement: S1 Checklist — (DOC) [file pgph.0001863.s001.doc]

STROBE Statement—checklist of items that should be included in reports of observational studies

|  | Item No | Recommendation |
| --- | --- | --- |
| **Title and abstract** | 1 | (*a*) Indicate the study’s design with a commonly used term in the title or the abstract  **Line 27** |
|  |  | (*b*) Provide in the abstract an informative and balanced summary of what was done and what was found |
| Introduction | | |
| Background/rationale | 2 | Explain the scientific background and rationale for the investigation being reported |
| Objectives | 3 | State specific objectives, including any prespecified hypotheses **Line 103-105** |
| Methods | | |
| Study design | 4 | Present key elements of study design early in the paper **Lines 110-117** |
| Setting | 5 | Describe the setting **(lines 78-87)**, locations, and relevant dates (**lines 112-113**), including periods of recruitment, exposure, follow-up, and data collection |
| Participants | 6 | (*a*) *Case-control study*—Give the eligibility criteria **Lines 111-114**, and the sources and methods of case ascertainment and control selection. Give the rationale for the choice of cases and controls **Lines 100-103** |
|  |  | (*b*) *Case-control study*—For matched studies, give matching criteria and the number of controls per case **Not applicable** |
| Variables | 7 | Clearly define all outcomes, exposures, predictors, potential confounders, and effect modifiers. Give diagnostic criteria, if applicable **Lines 113-167, Table S1** |
| Data sources/ measurement | 8* | For each variable of interest, give sources of data and details of methods of assessment (measurement) **Lines 131-179**. Describe comparability of assessment methods if there is more than one group |
| Bias | 9 | Describe any efforts to address potential sources of bias **Lines 191-195** |
| Study size | 10 | Explain how the study size was arrived at. **This was a pilot study, sample size was determined by budget constraints and feasibility to inform future studies. Line 130.** |
| Quantitative variables | 11 | Explain how quantitative variables were handled in the analyses. If applicable, describe which groupings were chosen and why |
| Statistical methods | 12 | (*a*) Describe all statistical methods, including those used to control for confounding **Starting Line 180** |
|  |  | (*b*) Describe any methods used to examine subgroups and interactions **Starting line 191** |
|  |  | (*c*) Explain how missing data were addressed – **subjects excluded if missing data present for a given analysis** |
|  |  | (*d*) *Case-control study*—If applicable, explain how matching of cases and controls was addressed **Not applicable**  *Cross-sectional study*—If applicable, describe analytical methods taking account of sampling strategy **Not applicable** |
|  |  | (*e*) Describe any sensitivity analyses **Not applicable** |

Continued on next page

| Results | | |
| --- | --- | --- |
| Participants | 13* | (a) Report numbers of individuals at each stage of study—eg numbers potentially eligible, examined for eligibility, confirmed eligible, included in the study, completing follow-up, and analysed **Lines 204-208, 96 cases and 99 controls enrolled, 29 cases were lost to follow-up and 30 controls were lost.** |
|  |  | (b) Give reasons for non-participation at each stage **Line 207-208, unable to assess** |
|  |  | (c) Consider use of a flow diagram **Previously published** |
| Descriptive data | 14* | (a) Give characteristics of study participants (eg demographic, clinical, social) and information on exposures and potential confounders, **Some is previously published, see related manuscript, new data is presented in Table S3** |
|  |  | (b) Indicate number of participants with missing data for each variable of interest **Relevant numbers are indicated in each table.** |
|  |  |  |
| Outcome data | 15* |  |
|  |  | *Case-control study—*Report numbers in each exposure category, or summary measures of exposure, **Relevant numbers are indicated in each table.** |
|  |  |  |
| Main results | 16 | (*a*) Give unadjusted estimates and, if applicable, confounder-adjusted estimates and their precision (eg, 95% confidence interval). Make clear which confounders were adjusted for and why they were included **Figure 1 (unadjusted) and Table 4 plus footnote.** |
|  |  | (*b*) Report category boundaries when continuous variables were categorized **Not applicable** |
|  |  | (*c*) If relevant, consider translating estimates of relative risk into absolute risk for a meaningful time period **Not applicable** |
| Other analyses | 17 | Report other analyses done—eg analyses of subgroups and interactions, and sensitivity analyses |
| Discussion | | |
| Key results | 18 | Summarise key results with reference to study objectives, **Line 305, 326, and 332** |
| Limitations | 19 | Discuss limitations of the study, taking into account sources of potential bias or imprecision. Discuss both direction and magnitude of any potential bias **Line 330, 350-358** |
| Interpretation | 20 | Give a cautious overall interpretation of results considering objectives, limitations, multiplicity of analyses, results from similar studies, and other relevant evidence. **Line 359-361** |
| Generalisability | 21 | Discuss the generalisability (external validity) of the study results, **Lines 306-308, 319-321, 328-331, 3333-335, 337-338** |
| Other information | | |
| Funding | 22 | Give the source of funding and the role of the funders for the present study and, if applicable, for the original study on which the present article is based, **Office of the Vice Chancellor or Research, Washington University** |

*Give information separately for cases and controls in case-control studies and, if applicable, for exposed and unexposed groups in cohort and cross-sectional studies.

**Note:** An Explanation and Elaboration article discusses each checklist item and gives methodological background and published examples of transparent reporting. The STROBE checklist is best used in conjunction with this article (freely available on the Web sites of PLoS Medicine at http://www.plosmedicine.org/, Annals of Internal Medicine at http://www.annals.org/, and Epidemiology at http://www.epidem.com/). Information on the STROBE Initiative is available at www.strobe-statement.org.
