# Supplementary material for: Prevalence of diarrheagenic Escherichia coli and impact on child health in Cap-Haitien, Haiti
Source: PLOS Glob Public Health. 2023 May 5;3(5):e0001863. doi: 10.1371/journal.pgph.0001863 (PMC10162540; doi:10.1371/journal.pgph.0001863)
Supplement: S1 Table — (DOCX) [file pgph.0001863.s002.docx]

**S1 Table. Definitions of survey variables.**

| Survey term | Definition |
| --- | --- |
| Currently breastfeeding, % | At time of survey administration, child was still breastfeeding (yes or no) |
| Times breastfeed in last 24h | Number of times in the last 24 hours (day or night) that the child breastfed |
| Animal source foods, % | Child was fed any animal sourced foods in the past 24 hours (yes or no) |
| Eggs, % | Child was fed any eggs in the past 24 hours (yes or no) |
| Suppressed appetite, % | Caregiver reported loss of appetite in the past 14 days (yes or no) |
| Nasal congestion/rhinorrhea, % | Child had nasal congestion or rhinorrhea in the past 14 days (yes or no) |
| Respiratory symptoms, % | Child had wheezing, difficulty breathing, and/or a cough in the past 14 days (yes or no) |
| Rash, % | Rash on the child in the past 14 days (yes or no) |
| Household Occupants | Total number of adults and children living in the household |
| Monthly household income >2000 Haitian Dollars, % | Total monthly income for all occupants in the household |
| Use bottled water, % | The household typically uses bottled water for drinking (yes or no) |
| Household with electricity, % | The household sometimes or always has electricity (yes or no) |
| Material floor is rock or dirt, % | The floor of the household is primarily made of rocks and dirt |
| Utilize flush toilet, % | Household uses a flush toilet (yes or no) |
