# Supplementary material for: Prevalence of diarrheagenic Escherichia coli and impact on child health in Cap-Haitien, Haiti
Source: PLOS Glob Public Health. 2023 May 5;3(5):e0001863. doi: 10.1371/journal.pgph.0001863 (PMC10162540; doi:10.1371/journal.pgph.0001863)
Supplement: S2 Table — (DOCX) [file pgph.0001863.s003.docx]

**S2 Table. Primers and target genes used in polymerase chain reactions.**

| **Pathogen** | **Primer** | **Target Gene** | **Primer Sequences** | Amplicon (bp) | Reference |
| --- | --- | --- | --- | --- | --- |
| EPEC | BFPA-F | *bfpA* | GGAAGTCAAATTCATGGGGG | 367 | [1] |
|  | BFPA-R |  | GGAATCAGACGCAGACTGGT |  |  |
|  | EAE-F | *eae* | CCCGAATTCGGCACAAGCATAAGC | 881 | [1] |
|  | EAE-R |  | CCCGGATCCGTCTCGCCAGTATTCG |  |  |
| EAEC | CVD432F | *aatA* | CTGGCGAAAGACTGTATCAT | 630 | [1] |
|  | CVD432R |  | CAATGTATAGAAATCCGCTGTT |  |  |
|  | AAIC F | *aaiC* | ATTGTCCTCAGGCATTTCAC | 215 | [1] |
|  | AAIC R |  | ACGACACCCCTGATAAACAA |  |  |
|  | AAF/I F | *aagA* | TCTATCTRGGGGGGCTAACGCTb | 220 | [2] |
|  | AAF/I R |  | ACCTGTTCCCCATAACCAGACCb |  |  |
|  | AAF/II F | *aaf* | CTACTTTATTATCAAGTGGAGCCGCTAb | 289 | [2] |
|  | AAF/II R |  | GGAGAGGCCAGAGTGAATCCTGb |  |  |
|  | AAF/III F | *aag3A* | CCAGTTATTACAGGGTAACAAGGGAAb | 370 | [2] |
|  | AAF/III R |  | TTGGTCTGGAATAACAACTTGAACG |  |  |
|  | AAF/IV F | *aag4A* | TGAGTTGTGGGGCTAYCTGGAb | 169 | [2] |
|  | AAF/IV R |  | CACCATAAGCCGCCAAATAAGCb |  |  |
| ETEC | LT-F | *eltB* | ACGGCGTTACTATCCTCTC | 273 | [3] |
|  | LT-R |  | TGGTCTCGGTCAGATATGTG |  |  |
|  | STp-F | *estB* | TCTTTCCCCTCTTTTAGTCAG | 166 | [3] |
|  | STp-R |  | ACAGGCAGGATTACAACAAAG |  |  |
|  | STh-F | *estA* | TACAAGCAGGATTACAACAC | 64 | [3] |
|  | STh-R |  | AGTGGTCCTGAAAGCATG |  |  |
|  | EatA-F | *eatA* | ACGGCGTTACTATCCTCTC | 1943 | [3] |
|  | EatA-R |  | ATATCCAGTCAGCACCCACT |  |  |
|  | EtpA-F | *etpA* | GGTTCAGGCAGTATCCAGAC | 999 | [3] |
|  | EtpA-R |  | GGTGTAGCTGTCTGACCACA |  |  |
|  |  |  |  |  |  |
|  |  |  |  |  |  |

EAEC, enteroaggregative *Escherichia coli*; EPEC, enteropathogenic *Escherichia* *coli*; ETEC*,* enterotoxigenic *Escherichia coli*

**References**

1. Liu J, Gratz J, Amour C, Nshama R, Walongo T, Maro A, et al. Optimization of Quantitative PCR Methods for Enteropathogen Detection. PLoS One. 2016;11(6):e0158199. doi: 10.1371/journal.pone.0158199. PubMed PMID: 27336160; PubMed Central PMCID: PMCPMC4918952.

2. Boisen N, Scheutz F, Rasko DA, Redman JC, Persson S, Simon J, et al. Genomic characterization of enteroaggregative Escherichia coli from children in Mali. J Infect Dis. 2012;205(3):431-44. doi: 10.1093/infdis/jir757. PubMed PMID: 22184729; PubMed Central PMCID: PMCPMC3256949.

3. Kuhlmann FM, Martin J, Hazen TH, Vickers TJ, Pashos M, Okhuysen PC, et al. Conservation and global distribution of non-canonical antigens in Enterotoxigenic Escherichia coli. PLoS Negl Trop Dis. 2019;13(11):e0007825. doi: 10.1371/journal.pntd.0007825. PubMed PMID: 31756188; PubMed Central PMCID: PMCPMC6897418.
