# Supplementary material for: Prevalence of diarrheagenic Escherichia coli and impact on child health in Cap-Haitien, Haiti
Source: PLOS Glob Public Health. 2023 May 5;3(5):e0001863. doi: 10.1371/journal.pgph.0001863 (PMC10162540; doi:10.1371/journal.pgph.0001863)
Supplement: S3 Table — (DOCX) [file pgph.0001863.s004.docx]

**S3 Table. Baseline characteristics based on study completion.**

|  | Completed study | N | Lost to follow-up | N | p-value^b^ |
| --- | --- | --- | --- | --- | --- |
| Child |  |  |  |  |  |
| Age, mo^a^ | 18.6 | 136 | 16.4 | 59 | 0.074 |
| Sex, % female | 55.1 | 136 | 45.8 | 59 | 0.294 |
| Dietary Intake |  |  |  |  |  |
| Currently breastfeeding, % | 53.6 | 125 | 59.3 | 54 | 0.593 |
| Times breastfeed 24h^a^ | 14.4 | 66 | 15.6 | 32 | 0.234^c^ |
| Animal source foods, % | 60.3 | 136 | 62.7 | 59 | 0.874 |
| Eggs, % | 17.6 | 131 | 15.5 | 58 | 0.730 |
| Morbidities, 14-d recall |  |  |  |  |  |
| Vomiting, % | 26.3 | 133 | 20.3 | 59 | 0.48 |
| Suppressed appetite, % | 42.5 | 134 | 47.5 | 59 | 0.633 |
| Nasal congestion/rhinorrhea, % | 52.9 | 136 | 57.6 | 59 | 0.639 |
| Respiratory symptoms, % | 52.2 | 136 | 69.5 | 59 | ***0.037*** |
| Rash, % | 22.4 | 134 | 20.3 | 59 | 0.898 |
| Fever (>38.0°C), % | 3.1 | 132 | 5.2 | 58 | 0.477 |
| Vaccinations received, % |  |  |  |  |  |
| Polio | 98.3 | 116 | 100 | 50 | 1.0^d^ |
| Rotavirus | 92.4 | 118 | 90 | 50 | 0.715^d^ |
| Typhoid | 45.9 | 111 | 33.3 | 48 | 0.334 |
| Maternal |  |  |  |  |  |
| Maternal age, y^a^ | 29.8 | 136 | 27.6 | 59 | 0.12^c^ |
| Secondary school and higher, % | 50 | 134 | 47.5 | 59 | 0.866 |
| Household |  |  |  |  |  |
| Household occupancy (N)^a^ | 6 | 132 | 5.7 | 57 | 0.41 |
| Drinking water bottled, % | 88.2 | 136 | 88.1 | 59 | 1 |
| Electricity, % | 30.1 | 136 | 44.8 | 58 | 0.071 |
| Material floor is rock or dirt, % | 12.6 | 135 | 23.7 | 59 | 0.083 |
| Flush toilet, % | 5.9 | 135 | 10.2 | 59 | 0.366^d^ |
| Households sharing toilet (N)^a^ | 2.8 | 46 | 2.5 | 15 | 0.44 |
| Pathogenic *E. coli* detection, % |  |  |  |  |  |
| ST ETEC OR ST-LT ETEC | 6.7 | 136 | 15.3 | 59 | 0.350 |
| LT ETEC | 11.1 | 136 | 6.8 | 59 | 0.058 |
| EAEC | 26.5 | 136 | 33.9 | 59 | 0.378 |
| tEPEC | 2.9 | 136 | 10.2 | 59 | 0.07^d^ |

^a^ Values are means ± standard deviations (SD)

^b^ statistical significance determined by either student t-test or Chi-squared, except where indicated.

^c^ Statistical significance determined by Mann-Whitney U test or ^d^Fisher’s exact test

EAEC, enteroaggregative *Escherichia coli*; LT ETEC*,* heat-labile enterotoxin enterotoxigenic *Escherichia coli*; ST ETEC, heat-stable enterotoxin enterotoxigenic *Escherichia coli*; tEPEC, typical enteropathogenic *Escherichia coli*
