# Supplementary material for: Prevalence of diarrheagenic Escherichia coli and impact on child health in Cap-Haitien, Haiti
Source: PLOS Glob Public Health. 2023 May 5;3(5):e0001863. doi: 10.1371/journal.pgph.0001863 (PMC10162540; doi:10.1371/journal.pgph.0001863)
Supplement: S4 Table — (DOCX) [file pgph.0001863.s005.docx]

**S4 Table.** **Additional details for Table 1**

| Column | 1 | | 2 | | 3 | | 4 | |  |
| --- | --- | --- | --- | --- | --- | --- | --- | --- | --- |
| Case or Control | Control (asymptomatic) | | Control (asymptomatic) | | Case (symptomatic) | | Case (symptomatic) | |  |
| Follow up symptoms | Asymptomatic | | Symptomatic | | Asymptomatic | | Symptomatic | |  |
|  | Mean (SD) or % | N | Mean (SD) or % | N | Mean (SD) or % | N | Mean (SD) or % | N | p-value^b^ |
| Child |  |  |  |  |  |  |  |  |  |
| ^a^Age, mo | 20.4 (7.6) | 45 | 17.6 (9.2) | 24 | 18.2 (7.6) | 41 | 17.1 (7.6) | 26 | 0.29 |
| Sex, % female | 66.7 | 30/45 | 50.0 | 12/24 | 41.5 | 17/41 | 61.5 | 16/26 | 0.10 |
| Dietary Intake in last 24 hours |  |  |  |  |  |  |  |  |  |
| ^g^Currently breastfeeding, % | 46.5 | 20/43 | 52.4 | 11/21 | 47.4 | 18/38 | 78.3 | 18/23 | 0.07 |
| ^a,e^Number breastfeeding episodes in a day | 13.1 (6.3) | 20 | 16.5 (4.9) | 11 | 13.9 (7.1) | 17 | 15.0 (5.6) | 18 | 0.48 |
| Animal source foods, % | 60 | 27/45 | 66.7 | 16/24 | 58.5 | 24/41 | 57.7 | 15/26 | 0.91 |
| ^g^Eggs, % | 16.3 | 7/43 | 20.8 | 5/24 | 15 | 6/40 | 20.8 | 5/24 | 0.90^d^ |
| Morbidities, 14-d recall, % |  |  |  |  |  |  |  |  |  |
| ^g^Vomiting | 13.6 | 6/44 | 31.8 | 7/22 | 41.5 | 17/41 | 19.2 | 5/26 | **0.02** |
| ^g^Suppressed appetite | 28.9 | 13/44 | 36.4 | 8/22 | 51.2 | 21/41 | 57.7 | 15/26 | 0.06 |
| Rhinorrhea | 51.1 | 23/45 | 50 | 12/24 | 48.8 | 20/41 | 65.4 | 17/26 | 0.56 |
| Cough, wheeze, or difficulty breathing | 44.4 | 20/45 | 58.3 | 14/24 | 53.7 | 22/41 | 57.7 | 15/26 | 0.62 |
| ^g^Rash | 22.2 | 10/45 | 27.3 | 6/22 | 17.1 | 7/41 | 26.9 | 7/26 | 0.71^d^ |
| ^g^Fever (>38.0°C) | 0 | 0/43 | 4.3 | 1/23 | 4.9 | 2/41 | 4 | 1/25 | 0.44^d^ |
| Vaccinations received, % |  |  |  |  |  |  |  |  |  |
| ^g^Polio | 100 | 41/41 | 100 | 22/22 | 96.8 | 40/41 | 95.5 | 25/26 | 0.42^d^ |
| ^g^Rotavirus | 97.6 | 41/42 | 82.6 | 19/23 | 93.3 | 28/30 | 91.3 | 21/23 | 0.14^d^ |
| ^g^Typhoid | 48.7 | 19/39 | 34.8 | 8/23 | 53.3 | 16/30 | 42.1 | 8/19 | 0.62^d^ |
| Anthropometric z scores |  |  |  |  |  |  |  |  |  |
| ^a^HAZ | -1.1 (1.1) | 45 | -1.4 (1.7) | 24 | -1.2 (1.0) | 41 | -1.3 (1.4) | 26 | 0.89 |
| ^a^WAZ | -0.90 (1.1) | 45 | -0.73 (1.4) | 24 | -0.89 (0.89) | 41 | -1.3 (1.3) | 26 | 0.27 |
| ^a^WHZ | -0.43 (1.1) | 45 | 0.04 (0.98) | 24 | -0.38 (0.87) | 41 | -0.89 (1.2) | 26 | **0.048**^c^ |
| Maternal |  |  |  |  |  |  |  |  |  |
| ^a^Maternal age, y | 31.5 (9.5) | 45 | 28.5 (7.4) | 24 | 29.8 (8.9) | 39 | 28.1 (8.7) | 26 | 0.37^c^ |
| Secondary school and higher, % | 55.6 | 25/45 | 45.8 | 11/24 | 48.7 | 19/39 | 46.2 | 12/26 | 0.83 |
| Household |  | 0/45 |  | 0/24 |  | 0/41 |  | 0/26 |  |
| ^a,g^Household occupants (N) | 6.1 (2.2) | 42 | 5.4 (1.7) | 24 | 6.0 (2.0) | 41 | 6.4 (2.4) | 26 | 0.35 |
| Use bottled water, % | 88.9 | 40/45 | 83.3 | 20/24 | 87.8 | 36/41 | 92.3 | 24/26 | 0.80^d^ |
| Electricity in home, % | 20 | 9/45 | 16.7 | 4/24 | 41.5 | 17/41 | 42.3 | 11/26 | **0.04** |
| ^g^Dirt or rock flooring, % | 2.3 | 1/44 | 8.3 | 2/24 | 17.1 | 7/41 | 26.9 | 7/26 | **0.01^d^** |
| ^g^Utilize flush toilet, % | 9.1 | 4/44 | 12.5 | 3/24 | 0 | 0/41 | 3.8 | 1/26 | 0.09^d^ |
| ^a,f^Households sharing toilet (N) | 2.5 (0.96) | 19 | 3.4 (1.8) | 9 | 2.4 (1.1) | 9 | 3.2 (0.92) | 9 | 0.18^c^ |
| Pathogenic *E. coli* detection, % |  |  |  |  |  |  |  |  |  |
| ST ETEC or ST-LT ETEC | 4.4 | 2/45 | 4.2 | 1/24 | 7.3 | 3/41 | 11.5 | 3/26 | 0.69^d^ |
| LT ETEC | 8.9 | 4/45 | 16.7 | 4/24 | 7.3 | 3/41 | 15.4 | 4/26 | 0.53^d^ |
| EAEC | 22.2 | 10/45 | 25 | 6/24 | 31.7 | 13/41 | 26.9 | 7/26 | 0.79^d^ |
| tEPEC | 2.2 | 1/45 | 0 | 0/24 | 4.9 | 2/41 | 3.8 | 1/26 | 0.77^d^ |
| aEPEC | 24.4 | 11/45 | 8.3 | 2/24 | 22.0 | 9/41 | 19.2 | 5/26 | 0.44 |

^a^ Values are means ± standard deviations (SD).

^b^ One-way ANOVA tests were used for continuous variables, chi-squared tests for categorical variables, unless otherwise indicated. Statistical significance indicated for p<0.05 in bold.

^c^ Significance of continuous variables assessed using Kruskal-Wallis test, or ^d^Fisher’s exact test.

^e^20 respondents for column 1, 11 for column 2, 17 for column 3, and 18 for column 4.

^f^19 respondents for column 1, 9 for all other columns,

^g^Number of respondents differs from Max N, see Table S4 for details

EAEC, enteroaggregative *Escherichia* *coli*; LT ETEC*,* heat-labile enterotoxin enterotoxigenic *Escherichia coli*; ST ETEC, heat-stable enterotoxin enterotoxigenic *Escherichia coli*; tEPEC, typical enteropathogenic *Escherichia* *coli*
