# Supplementary material for: Prevalence of diarrheagenic Escherichia coli and impact on child health in Cap-Haitien, Haiti
Source: PLOS Glob Public Health. 2023 May 5;3(5):e0001863. doi: 10.1371/journal.pgph.0001863 (PMC10162540; doi:10.1371/journal.pgph.0001863)
Supplement: S5 Table — (DOCX) [file pgph.0001863.s006.docx]

**S5 Table. Identification of multiple pathogenic *E.* *coli* by symptoms.**

|  | **Baseline** | |  |  | **Follow-up** | |  |  |
| --- | --- | --- | --- | --- | --- | --- | --- | --- |
|  | **Symptomatic (n=96)** | **Asymptomatic (n=99)** | **Total** | **p-value^a^** | **Symptomatic (n=50)** | **Asymptomatic (n=86)** | **Total** | **p-value^a^** |
| No E. *coli* detected | 44.8 | 55.6 | 50.3 | 0.133 | 54.2 | 55.4 | 55.0 | 0.889 |
| EAEC only | 21.9 | 18.2 | 20.0 | 0.519 | 10.4 | 18.1 | 15.3 | 0.240 |
| EPEC only | 6.3 | 6.1 | 6.2 | 0.956 | 8.3 | 2.4 | 4.6 | 0.118 |
| ETEC only | 12.5 | 9.1 | 10.8 | 0.443 | 14.6 | 8.3 | 10.7 | 0.272 |
| EAEC+EPEC | 5.2 | 4.0 | 4.6 | 0.746^b^ | 10.4 | 3.6 | 6.1 | 0.100^b^ |
| EAEC + ETEC | 1.0 | 4.0 | 2.6 | 0.369^b^ | 2.1 | 9.6 | 6.9 | 0.153^b^ |
| EPEC + ETEC | 6.3 | 2.0 | 4.1 | 0.167^b^ | 0 | 1.2 | 0.8 | 1^b^ |
| EAEC + EPEC + EPEC | 2.1 | 1.0 | 1.5 | 0.619^b^ | 0 | 1.2 | 0.8 | 1^b^ |

^a^ Chi-squared testing unless ^b^Fisher’s exact test compare symptomatic vs. asymptomatic participants.

EAEC, enteroaggregative Escherichia *coli*; EPEC, enteropathogenic Escherichia *coli*; ETEC*,* enterotoxigenic Escherichia *coli*
