# Supplementary material for: Prevalence of diarrheagenic Escherichia coli and impact on child health in Cap-Haitien, Haiti
Source: PLOS Glob Public Health. 2023 May 5;3(5):e0001863. doi: 10.1371/journal.pgph.0001863 (PMC10162540; doi:10.1371/journal.pgph.0001863)
Supplement: S6 Table — (DOCX) [file pgph.0001863.s007.docx]

**S6 Table. Diarrheagenic *E. coli* during rainy or dry seasons**

|  | **Symptomatic****^a^** | | |  | **Asymptomatic^a^** | | |
| --- | --- | --- | --- | --- | --- | --- | --- |
|  | Wet  (n=73) | Dry  (n=70) | p-value ^b^ |  | Wet  (n=93) | Dry  (n=86) | p-value ^b^ |
| ETEC | 27.4 | 12.9 | **0.031** |  | 23.7 | 12.8 | 0.061 |
| EAEC | 34.2 | 21.4 | 0.088 |  | 24.7 | 34.9 | 0.137 |
| EPEC | 5.5 | 2.7 | 0.951 |  | 6.5 | 0.0 | **0.029**^c^ |

^a^ Each time point was included independently

^b^ Chi-squared testing, unless otherwise indicated as ^c^Fisher’s Exact test

EAEC, enteroaggregative *Escherichia* *coli*; EPEC, enteropathogenic *Escherichia* *coli*; ETEC*,* enterotoxigenic *Escherichia coli*
