# Supplementary material for: Prevalence of diarrheagenic Escherichia coli and impact on child health in Cap-Haitien, Haiti
Source: PLOS Glob Public Health. 2023 May 5;3(5):e0001863. doi: 10.1371/journal.pgph.0001863 (PMC10162540; doi:10.1371/journal.pgph.0001863)
Supplement: S7 Table — (DOCX) [file pgph.0001863.s008.docx]

**S7 Table. Nutritional biomarker concentrations relative to total DEC detected.**

| Number of DEC | 0 | 1 | 2 | 3 |  |
| --- | --- | --- | --- | --- | --- |
| Number of participants | n=21 | n=20 | n=6 | n=2 | p-value ^b^ |
| Plasma DHA (µg/ml) ^a^ | 1.0 (0.34) | 1.0 (0.45) | 1.3 (0.54) | 0.83 (0.36) | 0.385 |
| Plasma choline (µg/ml) ^a^ | 4.2 (1.9) | 4.7 (2.6) | 5.3 (1.6) | 2.4 (0.27) | 0.380 |
| Plasma betaine (µg/ml) ^a^ | 7.0 (2.0) | 7.9 (3.3) | 9.6 (3.8) | 5.9 (2.1) | 0.170 |

^a^ Values presented are means and standard deviations (SD)

^b^ Statistical significance determined using ANOVA.

DHA, docosahexaenoic acid
