# Supplementary material for: Prevalence of diarrheagenic Escherichia coli and impact on child health in Cap-Haitien, Haiti
Source: PLOS Glob Public Health. 2023 May 5;3(5):e0001863. doi: 10.1371/journal.pgph.0001863 (PMC10162540; doi:10.1371/journal.pgph.0001863)
Supplement: S8 Table — (DOCX) [file pgph.0001863.s009.docx]

**S8 Table.** **Multivariable linear regression models with change in anthropometry as outcome and *E. coli* subtypes at baseline**

|  | **Change in HAZ^1^** | | | **Change in WAZ^2^** | | | **Change in WHZ^3^** | | |
| --- | --- | --- | --- | --- | --- | --- | --- | --- | --- |
|  | Coefficient B (SE) | p-value | Adjusted R^2^ | Coefficient B (SE) | p-value | Adjusted R^2^ | Coefficient B (SE) | p-value | Adjusted R^2^ |
| ETEC all | -0.15 (0.12) | 0.193 | -0.04 | -0.04 (0.12) | 0.736 | -0.02 | 0.001 (0.18) | 0.993 | -0.03 |
| ETEC ST or ST/LT | 0.03 (0.18) | 0.860 | -0.05 | 0.15 (0.19) | 0.426 | -0.02 | 0.23 (0.28) | 0.402 | 0.002 |
| ETEC LT | -0.26 (0.15) | 0.076 | -0.03 | -0.15 (0.15) | 0.299 | -0.02 | -0.14 (0.22) | 0.517 | -0.4e-6 |
| EPEC all | 0.13 (0.10) | 0.217 | -0.04 | 0.14 (0.11) | 0.187 | -0.01 | 0.11 (0.16) | 0.496 | 0.0003 |
| tEPEC | 0.19 (0.25) | 0.450 | -0.05 | 0.27 (0.27) | 0.326 | -0.02 | 0.19 (0.41) | 0.636 | -0.002 |
| aEPEC | 0.10 (0.11) | 0.334 | -0.05 | 0.11 (0.11) | 0.330 | -0.02 | 0.09 (0.17) | 0.605 | -0.001 |
| EAEC aata or aaic | 0.12 (0.09) | 0.166 | -0.04 | -0.01 (0.09) | 0.941 | -0.03 | -0.11 (0.14) | 0.434 | 0.001 |
| EAEC aata and aaic | 0.15 (0.10) | 0.127 | -0.03 | 0.04 (0.10) | 0.688 | -0.02 | -0.08 (0.16) | 0.615 | -0.001 |
| DEC all | 0.07 (0.09) | 0.461 | -0.05 | 0.03 (0.10) | 0.793 | -0.02 | 0.002 (0.15) | 0.991 | -0.003 |

^1^Adjusted for case-control group, animal source food intake, number of children in the household, sex, breastfeeding, and access to electricity

^2^Adjusted for case-control group, animal source food intake, and number of children in the household

^3^Adjusted for case-control group, animal source food intake, number of children in the household, minimum dietary diversity score, and household dietary diversity score

Abbreviations: HAZ, height-for-age z score; WAZ, weight-for-age z score; WHZ, weight-for-height z score;
