# Supplementary material for: Prevalence of diarrheagenic Escherichia coli and impact on child health in Cap-Haitien, Haiti
Source: PLOS Glob Public Health. 2023 May 5;3(5):e0001863. doi: 10.1371/journal.pgph.0001863 (PMC10162540; doi:10.1371/journal.pgph.0001863)
Supplement: S9 Table — (DOCX) [file pgph.0001863.s010.docx]

**S9 Table.** **Logistic regression models with stunting, underweight, and wasting as outcome and *E. coli* subtypes at baseline**

|  | **Stunting^1^** | | | **Underweight^2^** | | | **Wasting^3^** | | |
| --- | --- | --- | --- | --- | --- | --- | --- | --- | --- |
|  | **aOR** | **95% CI** | **p-value** | **aOR** | **95% CI** | **p-value** | **aOR** | **95% CI** | **p-value** |
| ETEC all | 1.96 | 0.79 – 4.71 | 0.135 | 3.58 | 1.52 – 8.49* | 0.003 | 3.19 | 1.07 – 9.03* | 0.030 |
| ETEC ST or ST/LT | 2.54 | 0.77 – 7.96 | 0.113 | 3.98 | 1.3 – 12.29* | 0.016 | 5.51 | 1.44 – 19.86* | 0.009 |
| ETEC LT | 1.31 | 0.34 – 4.26 | 0.671 | 2.37 | 0.76 – 6.86 | 0.119 | 1.24 | 0.18 – 5.17 | 0.787 |
| EPEC all | 0.99 | 0.41 – 2.25 | 0.985 | 2.34 | 1.07 – 5.10* | 0.032 | 1.86 | 0.64 – 5.03 | 0.231 |
| tEPEC | 0.85 | 0.12 – 3.99 | 0.847 | 2.48 | 0.58 – 9.68 | 0.194 | 1.11 | 0.06 – 6.87 | 0.926 |
| aEPEC | 1.04 | 0.40 – 2.48 | 0.931 | 1.98 | 0.84 – 4.56 | 0.111 | 1.96 | 0.64 – 5.51 | 0.214 |
| EAEC aata or aaic | 1.89 | 0.89 – 4.14 | 0.101 | 1.67 | 0.79 – 3.56 | 0.179 | 1.94 | 0.73 – 5.53 | 0.194 |
| EAEC aata and aaic | 1.41 | 0.63 – 3.08 | 0.397 | 2.57 | 1.19 - 5.55* | 0.016 | 2.23 | 0.82 – 6.01 | 0.109 |
| DEC any | 1.25 | 0.58 – 2.84 | 0.576 | 1.99 | 0.89 – 4.81 | 0.108 | 2.36 | 0.80 – 8.69 | 0.148 |

^1^Adjusted for case-control group, animal source food intake, number of children in the household, sex, breastfeeding, and access to electricity

^2^Adjusted for case-control group, animal source food intake, and number of children in the household

^3^Adjusted for case-control group, animal source food intake, number of children in the household, minimum dietary diversity score, and household dietary diversity score

Abbreviations: aOR, adjusted odds ratio; CI, confidence interval
